# Bayesian Inference on Predictors of Sex of the Baby

**DOI:** 10.3389/fpubh.2016.00102

**Published:** 2016-05-24

**Authors:** Bruno Scarpa

**Affiliations:** ^1^Department of Statistical Sciences, University of Padua, Padua, Italy

**Keywords:** sex of the baby, Bayesian hierarchical model, aggregated Bernoulli, human fertility

## Abstract

It is well known that the sex ratio at birth is a biological constant, being about 106 boys to 100 girls. However couples have always wanted to know and decide in advance the sex of a newborn. For example, a large number of papers appeared connecting biometrical variables, such as length of follicular phase in the woman menstrual cycle or timing of intercourse acts to the sex of new baby. In this paper, we propose a Bayesian model to validate some of these theories by using an independent database. Results show that we could not show an effect of the follicular length on the sex of the baby. We also obtain a slightly larger probability, although not significant, of conceiving a female just after the mucus peak day.

## 1 Introduction

A number of researchers have been investigating if the biological system determining the sex of a newborn is completely random or if it could be predetermined. The starting point is clearly the fact that, although the proportions of sperm conducing chromosomes X and Y are the same, the number of male births is slightly higher than the number of females (1).

Many studies analyzed the relation between sex of the newborn and biological and social factors, such as length of follicular phase (2), pattern of intercourse (3–7), diet of the woman (8, 9), behavior during coital act (10), parents age (11, 12), historical events (11), atmospheric temperature (13), environmental pollution (14), and so on. Despite this very large set of researches in this field, we observed a lack of validation studies based on independent data. Most of these studies are based on really small samples and are difficult to replicate, by requiring specific and expensive epidemiological studies to be run.

In this paper, we are interested in evaluating the effect of two biometrical variables on the sex of the baby, which have been studied extensively in the past: the length of the follicular phase and the pattern of intercourse. Weinberg et al. (2) found that cycles with shorter follicular phase lengths are slightly more likely to result in male babies, while cycles with longer follicular phases are more likely to result in female babies. This theory, however, has been discussed by Gray et al. (15), concluding that there is no association between follicular phase length and sex ratio.

Even more debated is the other hypothesis we are analyzing. Kleegman (3) first supposed that if conception occurs in the day of ovulation the chances that the baby is a male are about 80%, while if the intercourse occurs 2 days earlier the same chances are given to a female baby. Some years later, in three different studies, Guerrero (4–6) shows that the probability to have a male is higher if intercourse occurs far from the ovulation. Billings and Westmore (7), supported by a study by McSweeney (16), instead, state that if intercourse occurs the day of ovulation or the following ones, the probability to have a male is higher, while if intercourse occurs early in the fertile window there are more chances to get a female. Other authors’ contribution to this debate were Shettles (10) and Perez et al. (17) each of them with his own position. From this short review, it is clear that different studies give completely different results, and we need an independent dataset to test such theories.

The validation or confutation of these theories is made difficult by the fact that, typically, multiple intercourse acts occur during a cycle, and it is not known which is the one responsible for the conception. As discussed in many papers [e.g., Ref. (18–21)], in such a case, a statistical model is needed to analyze available data, and exploit all cycles and not only the very small number of one-intercourse cycles.

The sex of the baby can be represented by the aggregation across Bernoulli trials for each intercourse day (22). Because of this aggregated Bernoulli data structure and the need to account for dependency among the multiple cycles from a woman, statistical analyses can be challenging to implement.

In this paper, we propose the use of a Bayesian model for the probability to conceive a female (or a male) and we evaluate the effect of day of intercourse and length of the follicular phase on the sex of the baby by using a large dataset obtained by the union of two fecundability studies led by Colombo (19, 20). We obtain posterior distributions of the parameters, by modifying the Gibbs sampling algorithm proposed by Dunson and Stanford (21) to model conception probabilities.

In Section 2, we present the dataset obtained from the two fecundability studies, Section 3 proposes the model and priors, Section 4 applies the model to data and Section 5 discusses the results. All the analyses in this paper have been implemented in R, a language and environment for statistical computing (23); code is available upon request to the author.

## 2 Materials and Methods

### 2.1 Two Studies of Daily Fecundability

In order to develop and fit a model relating biometrical variables and sex of the baby, it is necessary to have very complete data on biomarkers and intercourse days for a large number of menstrual cycles that ended up with a conception. Unfortunately, such data are difficult to obtain, and only a small number of available studies include detailed and reliable information on couples’ behaviors. Also, the majority of cycles collected in these studies do not end up with a conception, so the number of useful cases considered to evaluate sex of the baby in each study is quite small. For this reason, we focus on the union of data collected from two different studies, having quite a similar design and coordinated by the same researcher.

#### 2.1.1 European Fecundability Study

The European Study of Daily Fecundability (19) collected data on 782 couples, which were married or in a stable relation, from seven European centers (Milan, Verona, Lugano, Dusseldorf, Paris, London, and Brussels) providing services on fertility awareness and natural family planning by using symptothermal methods. Women enrolled were between 18 and 40 years of age, were not taking hormonal medications or drugs affecting fertility, had no known impairment of fecundity and had at least one menses after cessation of breastfeeding or after delivery and were experienced in the use of one method of natural family planning. The participants were followed prospectively during one or more menstrual cycles, as they recorded daily basal body temperatures, vaginal observations from cervical mucus, and the days during which intercourse and menstrual bleeding occurred. The women had received training at the study centers on how to identify different types of sensation and mucus. Teachers classified each day of the cycle according to a four-point scale according to the type of mucus described by women.

Additional 99 subjects from a different study (24, 25), carried out in Auckland (New Zealand), were included retrospectively in view of their relevance to the aims of the study. As discussed by Colombo and Masarotto (19) recruitment and instruction to the women was quite similar to the one for the European Study. The main specificity in this case was that study design restricted the couples to only one act of intercourse during the fertile phase of the cycle that is a suitable window of days around ovulation.

Data on a total of 7017 (6724 from the European centers and 293 from the New Zealand study) menstrual cycles have been collected, with 453 of these cycles (375 cycles from European centers and 78 from the New Zealand study) ending up in a conception. The average age in years of the women was 29.4 (SD = 4.15).

By using the available data, cervical mucus peak may be obtained as proxy for the ovulation day. The cervical mucus peak day was defined as the last day with best quality mucus by sensation or appearance, in a specific cycle of the woman, known retrospectively. This peak day was taken as “Mucus reference day” and identified as day 0. The number of days between the first day of menstruation and the mucus reference day for each cycle is a proxy for the length of the follicular phase. By considering this indicator, the average length of the follicular phase was, restricting the dataset to cycles conceiving a baby, 17.0 days (SD = 5.6), while the same quantity for cycles conceiving a male was 16.98 days (SD = 5.81) and for cycles conceiving a female was 17.01 days (SD = 5.40).

#### 2.1.2 Italian Fecundability Study

A second prospective study of fecundability (20) was led by the same researcher and carried out in four Italian centers providing services on fertility awareness and natural family planning by using the Billings ovulation method (26) and was concentrated in estimating the effect of cervical mucus symptoms on the probabilities of conception. This study enrolled 191 women providing data on 2536 menstrual cycles, with 141 of these cycles ending in a conception. Women enrolled were between 18 and 40 years of age, were married or in stable relationship, had at least one menses after cessation of breastfeeding or after delivery, were not taking hormonal medications or drugs affecting fertility, and were experienced in the use of the Billings ovulation method of natural family planning. The average age in years of the women was 29.96 (SD = 4.18). Also in this case, the participants were trained at the study centers on how to identify different types of sensation and mucus and collected detailed daily records of vulvar observations of the cervical mucus symptom, and recorded the days during which intercourse and menstrual bleeding occurred.

Teachers classified each day of the cycle according to a five-point scale according to the type of mucus symptom described by women and identified the Billings mucus peak defined as “the last day of the cycle during which at least one characteristic of high fertility in mucus type is observed or felt; this day must also be preceded by an adequate increase in sensations and the appearance of mucus characteristics, and followed by an abrupt change” (20). Ovulation is expected approximately within 2 days of the peak, and this day was used as a reference to determine the end of the fertile phase. The average length of the follicular phase was, restricting to cycles conceiving a baby, 17.24 days (SD = 6.49) and the same quantities for cycles conceiving a male was 17.20 days (SD = 7.21) and for cycles conceiving a female was 17.28 days (SD = 5.69).

**Figure 1:**
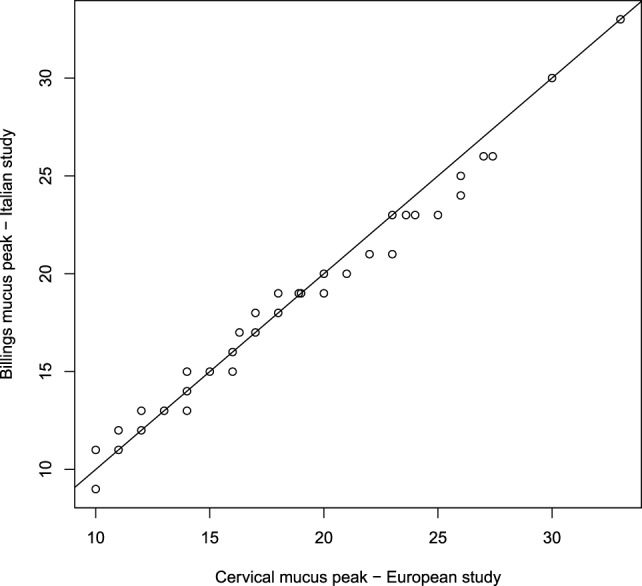
**qq-plot comparing the distributions of *cervical mucus peak* collected in the European study and the *Billings mucus peak* collected in the Italian study**.

The study designs of both, the European and the Italian Fecundability, studies are very similar and some analysis (not presented here) have shown that women enrolled in these different studies have reasonably similar characteristics. Since we are interested in identifying the fertile window by using the same criteria, the main problem in joining data from the two studies is related to the indicator for the day of ovulation used. Although in both studies an indicator based on cervical mucus is used, its definition is slightly different between the two studies, in particular because the classification of mucus (in the European study) and mucus symptoms (in the Italian study) are different according to the different methods of natural family planning. However, we analyzed and compared the distributions of the mucus peak, and consequently of the follicular phase, as estimated in the two studies by using in both cases mucus peaks, and we did not found significant differences. For example, the qqplot comparing the distributions of the cervical mucus peak and of Billings mucus peak collected by the two studies is plotted in Figure 1 and shows that the two distributions are not different. In a qqplot, the quantiles of two distributions are plotted: if these points are on the diagonal, this means that the two distributions are equal; the points out of the diagonal show some difference between the distributions. We concluded that the two indicators are similar enough to be considered as one. Note that similar analysis comparing the cervical mucus peak and other ovulation indicators, such as the rise in basal body temperature, shows significant differences (27, 28). We, therefore, considered as mucus peak (MP) the cervical mucus peak for the European study and the Billings mucus peak for the Italian study.

By joining the two studies and selecting only the cycles with conceptions for which the MP was observed, we obtained a total of 521 menstrual cycles that we analyzed in this paper. Table 1 shows the number of cycles with intercourse for each day in the fertile window for the joint dataset and its frequency over the total number of available cycles. Clearly, a cycle may show an intercourse act in more than 1 day.

**Table 1:** **Number of cycle with intercourse for each day in the fertile window and its relative frequency over the total number of available cycles (521)**.

Intercourse day	Absolute frequency	Relative frequency (%)
−8	106	20.34
−7	92	17.66
−6	124	23.80
−5	133	25.53
−4	153	29.37
−3	120	23.03
−2	165	31.67
−1	154	29.56
0	203	38.96
1	128	24.57
2	100	19.19
3	97	18.62

In addition to the day of intercourse, we are interested in analyzing the length of follicular phase as predictor of the sex of the baby, by testing the hypothesis discussed by Weinberg et al. (2). These authors show that the relation between sex ratio and follicular phase length is not linear and likely not even monotone. By using their results and to allow our model to include possible non-linearities, we analyze this relationship by considering four classes of follicular phase lengths: shorter of 13, between 13 and 16, between 16 and 19, and longer than 19 days. Table 2 shows the frequency distribution of the categorized follicular phase length.

**Table 2:** **Observed distribution, absolute and relative, of categorized follicular length**.

Follicular length (days)	Frequency	Percentage (%)
≤13	147	28.21
14–16	155	29.75
17–19	119	22.84
>19	100	19.19

Both of the data sets are available for researches complying with the objectives of the study design, which are stated in the website http://www.stat.unipd.it/en/fare-ricerca/basi-di-dati where, also, rules to have access to the data are explained.

### 2.2 The Bayesian Model and Priors

An intercourse act can result in conception only if it occurs in a mid-cycle window (29). Therefore, only intercourse acts during the fertile interval may impacts on the sex of the baby. Moreover, there may be multiple intercourse acts that occur during the potentially fertile phase of the cycle and we need a statistical model to allocate day-specific probabilities of conceiving a male or a female. In addition, we would like to evaluate the effect of covariates, such as the length of the follicular phase.

In order to predict conception probabilities, by considering what is typically done to predict probability of conception [e.g., Ref. (18–21)], we propose a model to predict the probabilities of conceiving a male or a female, which only considers cycles with conception. Let *Y_*i*_* be an indicator of the sex of the newborn (0 = male and 1 = female) in conception cycle *i* (*i* = 1, …, *n*), *X_*i*_* = [*X_*i*_*_1_, …, *X_*iK*_*]*^*T*^* a vector of intercourse indicators for days 1, …, *K* (sometimes it may be useful to index days relatively to the day of ovulation), and *U_*i*_* = [*u_*i*_*_1_, …, *u_*iH*_*]*^*T*^* a covariate *H*-vector for cycle *i*, in our case we consider the length of the follicular phase discretized in classes. The total number of parameters involved in the model is, therefore, the sum of *K* parameters related to the days of intercourse and *H* parameters related to other covariates possibly available (in our case three parameterizing the four classes of follicular length).

Following Barrett and Marshall (18), we assume independence of intercourse acts on different days and we replace their day-specific probability of conception with the day-specific probability of conceiving a male, only considering conceiving cycles. The probability of having a male for a given conceiving cycle is given by the probability of not conceiving a female. The probability of conceiving a female is the product of the probabilities of not conceiving a male in each day when an intercourse act was observed. Our model, therefore, is

(1) $$P(Y_{i}=0|X_{i},u_{i})=1-\prod_{k=1}^{K}\;{\left(1-q_{ik}\right)}^{X_{ik}}$$

where *q_*ik*_* is the day-specific probability of conceiving a male in cycle *i* given intercourse only on day *k*.

Following Dunson and Stanford (21), the relation between *q_*ik*_* and available covariates *U_*i*_* is modeled by

(2) $$q_{ik}=1-\text{exp}(-\text{exp}(v_{ik}^{T}\beta ))$$

where β = [β_1_, …, β*_*K*_*,β*_*K*_*_+1_, …, β*_*K*_*_+_*_*H*_*]^T^ is a vector of regression coefficients and *V_*i*_* = [*v_*i*_*_1_, …, *v_*ik*_*, *v_*ik*_*_+1_, …, *v_*iK*+*H*_*]*^*T*^* is a (*K* + *H*)-vector related to cycle *i*. We follow the same authors by considering the first *K* elements of vector *V_*i*_* as indicators of the day of intercourse observed in cycle *i*. Therefore, in this case, we define λ*_*k*_* = exp(β*_*k*_*) for *k* = 1, …, *K*, as baseline parameters indicating the day-specific probabilities of conceiving a female in each day of the fertile interval with an intercourse. The remaining *H* elements of *V_*i*_* are the covariates included in *U_*i*_* and *γ_h_* = exp(β*_*K*_*_+_*_*h*_*), *h* = 1, …, *H*, are the parameters characterizing the changes across levels of categorical covariates. In particular, if we consider as a covariate the length of follicular phase in classes, which is an ordered categorical predictor *z_*i*_* = 1, …, *d* (in our case *d* = 4), the parameters λ_1_, …, λ*_*K*_* are baseline parameters characterizing the distribution of *Y_*i*_* for subjects with *z_*i*_* = 0 for days *k* = 1, …, *K*, while *γ_h_* characterizes the multiplicative change in the probability to conceive a female attributable to increasing *z_*i*_* from *h* to *h* + 1. We, therefore, may re-write (2) as

$$p_{ik}=1-q_{ik}=\lambda_{k}\prod_{h=1}^{z_{i}-1}\;\gamma_{h},$$

where here *p_*ik*_* is the day-specific probability of conceiving a female in cycle *i* given intercourse only on day *k*.

Note that in this case, λ*_*k*_* is a parameter related to day-specific covariates, while *γ_h_* is a parameter of cycle-specific covariates, outlining that, differently from the other proposed models (1, 2) for sex of the baby, our model may include both day-specific and cycle-specific covariates, by also allowing cycles for multiple intercourse days.

The Bayesian approach for the estimate of the coefficients λ*_*k*_* and γ*_*h*_* require the specification of prior distributions for the parameters included in the model. As done by Dunson and Stanford (21) in a similar context, we chose gamma priors, which are conditionally conjugate for each of the parameters λ*_*k*_*’s and one-inflated gamma distributions as priors for the γ*_*h*_*’s, allowing for a positive probability of no effect of the cycle-specific covariates. The joint posterior distribution for all the parameters is summarized as follows

$$\begin{array}{l} \left\{\prod_{k=1}^{K}\;G(\lambda_{k};a_{0k},b_{0k})\right\}\left\{\prod_{h=1}^{d-1}\;\left(I_{1}-G(\gamma_{h};\pi_{h},a_{0h},b_{0h})\right)\right\}\;\prod_{i=1}^{n}\;\left(\prod_{k=1}^{K}\;{\left(\lambda_{k}\prod_{h=1}^{z_{i}-1}\;\gamma_{h}\right)}^{X_{ik}}\right) \end{array}$$

where *𝒢*(*a,b*) denotes the gamma density with mean *a*/*b* and variance *a*/*b*^2^ and *I*_1_ − *𝒢*(*γ_h_*; *π_h_*,*a*_0_*_*h*_*,*b*_0_*_*h*_*) the one-inflated gamma density consisting of the mixture of a point mass at one (with probability *π_h_*) and a *𝒢*(*a*_0_*_*h*_*,*b*_0_*_*h*_*) density. Posterior computation is based on the introduction of an auxiliary vector of independent Poisson latent variables following log-linear gamma frailty models, as already done in the algorithm proposed by Dunson and Stanford (21), and proceed with a Gibbs sampling algorithm (see Appendix). The specification of the covariate and of the priors for the prediction of the sex of the baby will be discussed in the next section.

## 3 Results

We used the model described in expression (1) to relate the length of follicular phase and the timing of intercourse in the fertile interval to the probability of conceiving a female. As fertile interval, following Colombo and Masarotto (19), we consider the 12 days window included between 8 days before and 3 days after MP. Therefore, we let

$$\begin{array}{l} U_{ik}=[I(k=1),...,I(k=12),I(13<w_{i}\leq 16),\;I(16<w_{i}\leq 19),I(w_{i}>19){]}^{T} \end{array}$$

where *k* indexes the day in the fertile interval, with *k* = 1 eight days prior to ovulation, identified by MP, and *k* = 12 three days after the identified day of ovulation. Here *w_*i*_* is the length of the follicular phase of the *i*th cycle and we considered changes across the four classes of follicular length, cycles shorter or equal to 13 days, between 14 and 16, between 17 and 19, and larger than 19. However, following Dunson and Stanford (21), we parameterize the follicular length classes by considering the increasing effect of passing from one length class to the following one. The regression coefficients are divided into two subvectors β = (β_1_, β_2_), with β_1_ characterizing the baseline changes in the probability of conception according to timing in the fertile interval, and β_2_, which includes three parameters characterizing the changes across the four classes of follicular length. For the values of *λ_k_* = exp(β_1,*k*_), for *k* = 1, …, 12, we chose diffuse priors by letting *a*_0_*_*k*_* = *b*_0_*_*k*_* = 1 for *k* = 1, …, 12 as hyper-parameters of the gamma distribution.

The exponentiated regression coefficients, *γ_h_* = exp(β_2,*h*_) for *h* = 1, 2, 3, measure changes in the probability of conceiving a female associated with the follicular length changing between classes. For these parameters by incorporating a point mass at *γ_h_* = 1.0 for *h* = 1, 2, 3, we assign positive prior probability to regions where different follicular length classes have the same effect on the probabilities of conceiving a female. Following the strategy of Westfall et al. (30), we let *π_h_* = 0.5^1/3^ in order to assign 0.5 prior probability to the null hypothesis of no association between the follicular length class and the day-specific probabilities to conceive a female. The choice *a*_0_*_*h*_* = *b*_0_*_*h*_* = 0.1 allows for a high degree of uncertainty in the values of *γ_h_* under the alternative hypothesis.

**Figure 2:**
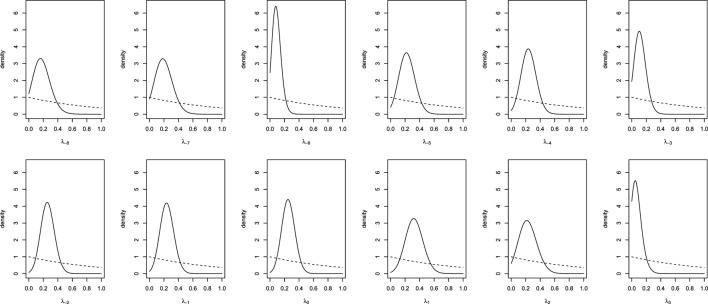
**Prior (dashed line) and posterior (solid lines) densities for day-specific probabilities of conceiving a female**.

To obtain results for the real data from the joined European and Italian studies, we run the MCMC analysis with a burn-in of 5,000 iterations and a collection interval of 40,000 iterations. Convergence was assessed by examining the stationarity of the distribution of the parameters. Trace-plots and standard diagnostic criteria (Geweke and Gelman and Rubin statistics) of all the parameters provided no evidence against convergence. Effective sample size was obtained for each parameters chain and all were larger than four thousand units. The prior and posterior densities for day-specific parameters (indexed with respect to the ovulation day, λ_−8_, …, λ_3_) are plotted in Figure 2. Although we suppose vague priors, the posteriors seem, for all parameters, relatively concentrated. In some days (e.g., days −7, 1, 2) the probability to conceive a female seems higher and in other days (e.g., days −6, −3, 3) it seems lower. However, all the posterior distributions show a relatively large variability (although much less than the prior) and all of them seem to assign a reasonable probability to values around 0.5 (or 0.48, the probability of generating a girl given the sex ratio at birth). It seems that there is not enough evidence to consider as significant the observed differences in probabilities.

**Figure 3:**
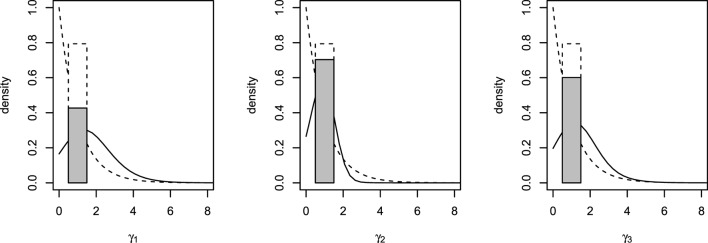
**Prior (dashed lines) and posterior (solid lines) densities for γ_1_, γ_2_, and γ_3_**. Posterior probability of γ*_h_* = 1 represented with a shaded rectangle.

Posterior summaries of the parameters are presented in Table 3, and the prior and posterior densities for the effect parameters of follicular length classes γ_1_, γ_2_, and γ_3_ are plotted in Figure 3. Each increase of one class in follicular length results in a non-significant increase or decrease in the probabilities of conceiving a female. In fact, the probability that each effect parameters is equal to one is around 50%, indicating that no increase in the effect is significant. Therefore, the follicular length seems not to have any effect on the probability to conceive a female.

**Table 3:** **Posterior summaries of day-specific parameters (λ’s) and of follicular length increasing parameters (γ’s)**.

Parameter	Mean	Median	SD	95% credible interval	$\hat{P}(\gamma_{h}=1)$
λ_−8_	0.58	0.57	0.19	(0.22, 0.94)	–
λ_−7_	0.68	0.70	0.19	(0.27, 0.98)	–
λ_−6_	0.22	0.21	0.11	(0.06, 0.46)	–
λ_−5_	0.54	0.54	0.13	(0.28, 0.78)	–
λ_−4_	0.53	0.53	0.11	(0.31, 0.74)	–
λ_−3_	0.23	0.22	0.10	(0.05, 0.45)	–
λ_−2_	0.47	0.47	0.09	(0.30, 0.64)	–
λ_−1_	0.42	0.42	0.09	(0.25, 0.60)	–
λ_0_	0.52	0.52	0.09	(0.34, 0.70)	–
λ_1_	0.68	0.69	0.10	(0.47, 0.87)	–
λ_2_	0.62	0.63	0.15	(0.29, 0.88)	–
λ_3_	0.17	0.14	0.12	(0.01, 0.46)	–
γ_1_	1.54	1.06	0.84	(0.79, 3.84)	0.43
γ_2_	0.82	1.00	0.38	(0.03, 1.07)	0.70
γ_3_	1.28	1.00	0.71	(0.33, 3.33)	0.60

New Zealand data included in the “European fecundability” data set are obtained from a study with a different protocol with respect to the other available data. However, as discussed in Colombo and Masarotto (19), data and results are quite comparable between New Zealand and European studies. Thus, in order to increase the sample size of our analysis, in particular to estimate precision of our estimates, we prefer to include these data in our analysis. However, a sensitivity analysis was performed to confirm the validity of our results by repeating the estimates without these data. The results show very little differences in the point estimates (λ_−8_ = 0.61, λ_−7_ = 0.57, λ_−6_ = 0.25, λ_−5_ = 0.45, λ_−4_ = 0.53, λ_−3_ = 0.25, λ_−2_ = 0.67, λ_−1_ = 0.47, λ_0_ = 0.50, λ_1_ = 0.81, λ_2_ = 0.53, λ_3_ = 0.19, γ_1_ = 1.23, γ_2_ = 0.86, γ_3_ = 1.20) which are all included in the credibility intervals shown in Table 3.

**Figure 4:**
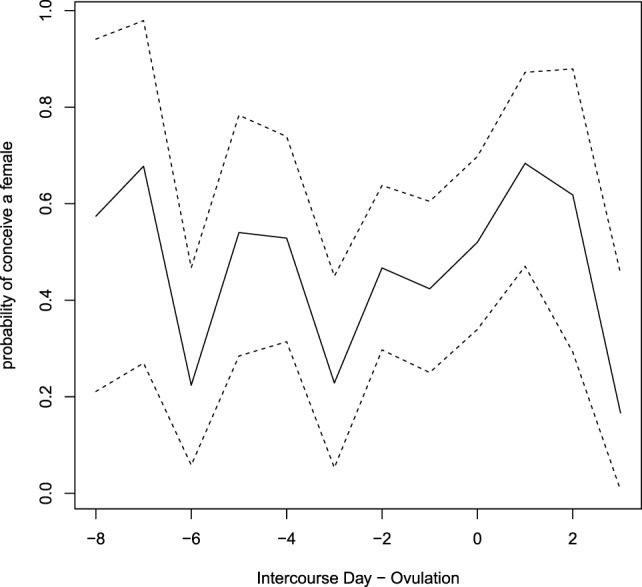
**Estimated day-specific probabilities of conceiving a female**. Dashed lines show credibility intervals.

The estimated day-specific sex of the babies probabilities with 95% credible intervals is plotted in Figure 4. Credibility intervals are quite large and although a large variability between days is visible, it seems that there are no clear pieces of evidence on days with higher (or lower) probability to conceive a female.

## 4 Discussion

This article has applied a Bayesian approach for inferences on predictors of day-specific probabilities of conceiving a female in the menstrual cycle. A variable selection-type prior is used for the regression coefficients, which allows uncertainty in the predictors to be included in the model and can be used to construct hypothesis tests comparing null hypotheses of no association with unrestricted alternatives.

Our analysis clearly show that we could not show an effect of the follicular length on the sex of the baby, once we consider the day-specific effect, by validating, using independent data, the results by Gray et al. (15).

The significance of the day-specific effect seems to be more difficult to be examined. Although our point estimates seem to show a pattern in the probabilities of conceiving a female, since posterior mode seems to be higher just after the mucus peak day, the variability of the posterior distribution, which reflect the variability in the data, does not allow us to give any evidence for these conclusions. In fact, credibility intervals are very large for each parameter and almost always include the sex ratio at birth (0.485). Therefore, it seems that, up to nowadays, we do not have any actual evidence to suggest different behaviors in the pattern of intercourse for people willing to conceive a female or a male.

## Ethics Statement

No vulnerable population was involved. Ethics committee: Institutional Review Board of the Fondazione Lanza (Padua, Italy).

## Author Contributions

BS is the sole author of this work.

## Conflict of Interest Statement

The author declares that the research was conducted in the absence of any commercial or financial relationships that could be construed as a potential conflict of interest.

## Appendix

Gibbs Sampling Algorithm for Posterior Computation

For posterior computation, following Dunson and Stanford (21), we first introduce for each units auxiliary variables

$$Z_{ik}∼\text{Poisson}(\text{exp}(u_{ik}^{T}\beta ))$$

such that

$$Y_{i}=1\left(\sum_{k=1}^{K}\;X_{ik}Z_{ik}>0\right)$$

where 1(*x*) is the indicator function. If we integrate out the auxiliary Poisson variables, which have no effect on parameter interpretation, we have that *Y_*i*_* = 0 if and only if *Z_*ik*_* = 0 for all *k* such that *X_*ik*_* = 1, that is there was an intercourse every day in the *i*th cycle.

Full conditional posterior distributions can then be obtained for each of the parameters and latent variables.

Step 1. Sample from the full conditional distribution of *Z_*i*_* = Σ*_*k*_*X*_*ik*_*Z*_*ik*_* by setting *Z_*i*_* = 0 if *Y_*i*_* = 0 and otherwise sampling sequentially from
  $\begin{array}{c} \pi (Z_{i}|Y_{i}=1,\beta ,\text{data})=\text{Poisson}(\sum_{k=1}^{K}\;X_{ik}p_{ik}) \end{array}$ truncated so that Z_i_ > 0,
  $$\begin{array}{c} \pi (Z_{i1},...,Z_{iK}|Z_{i},Y_{i}=1,\beta ,\text{data})=\text{Multinomial}\left(Z_{i};\frac{X_{i1}p_{i1}}{\sum_{k}\;X_{ik}p_{ik}},...,\frac{X_{iK}p_{iK}}{\sum_{k}\;X_{ik}p_{ik}}\right) \end{array}$$
  where *p_*i*_* = [*p*_1_, …, *p_*K*_*] and $p_{ik}=\text{exp}\left(u_{ik}^{T}\beta \right)=\lambda_{k}\prod_{h=1}^{z_{i}-1}\;\gamma_{h}$.
Step 2. Sample λ_1_, …, λ*_*K*_* from their conjugate full conditional distributions:
  $$\begin{array}{l} \pi (\lambda_{k}|Z_{\left[X=1\right]},\beta_{\left(-\lambda_{k}\right)},\text{data})=\text{Gamma}\left(\lambda_{k};a_{0k}+\sum_{i:X_{ik}=1}\;Z_{ik},b_{0k}+\sum_{i:X_{ik}=1}\;\prod_{h=1}^{z_{i}-1}\;\gamma_{h}\right), \end{array}$$
  where **Z**_[_**_**x**_**_=1]_ = *Z_*ik*_*: *X_*ik*_* = 1 and we integrate out *Z_*ik*_*: *X_*ik*_* = 0.
Step 3. Sample γ_1_, …, γ*_*H*_* from their conjugate full conditional distributions:
  $$\pi (\gamma_{k}|Z_{\left[X=1\right]},\beta_{\left(-\gamma_{k}\right)},\text{data})=I_{1}-\text{Gamma}(\gamma_{h};{\tilde{\pi }}_{h},{\tilde{a}}_{h},{\tilde{b}}_{h}),$$
  where ${\tilde{a}}_{h}=a_{h}+\sum_{i,k:X_{ik}=1}\;1_{\left(h<w_{ik}\right)}Z_{ik},\;{\tilde{b}}_{h}=b_{h}+\sum_{i,k:X_{ik}=1}\;1_{\left(h<z_{i}\right)}\lambda_{k}\prod_{j:j\neq h}^{z_{i}-1}\;\gamma_{j},$ and
  $$\begin{array}{l} {\tilde{\pi }}_{h}=\frac{\pi_{0h}\text{exp}(-\sum_{i,k:X_{ik}=1}\;1_{\left(h<z_{i}\right)}\lambda_{k}\prod_{j:j\neq h}^{z_{i}-1}\;)}{\pi_{0h}\text{exp}(-\sum_{i,k:X_{ik}=1}\;1_{\left(h<z_{i}\right)}\lambda_{k}\prod_{j:j\neq h}^{z_{i}-1}\;)+(1-\pi_{0h})\frac{C(a_{h},b_{h})}{C({\tilde{a}}_{h},{\tilde{b}}_{h})}}. \end{array}$$
  where *C*(*a,b*) = *b^*a*^*/Γ(*a*).

Repeat steps 1–3 until apparent convergence and calculate posterior summaries based on a large number of additional iterations.
